# An international cohort study of wound closure and surgical site infection prevention strategies in abdominal surgery (WOLVERINE): Protocol for a multicenter international study

**DOI:** 10.1111/codi.70364

**Published:** 2026-02-13

**Authors:** European Society of Coloproctology (ESCP) WOLVERINE Collaborating Group, Alaa El‐Hussuna, Sue Blackwell, Sanjay Chaudhri, Sharfuddin Chowdury, Dragomir Dardanov, Audrius Dulskas, Muhammed Elhadi, Caterina Foppa, Matteo Frasson, Gaetano Gallo, James Glasbey, Michael Kelly, Elizabeth Li, Ana Maria Minaya Bravo, Dion Morton, Peter Neary, Ionut Negoi, Francesco Pata, Gianluca Pellino, Thomas Pinkney, Gabrielle H. van Ramshorst, Shaji Sebastian, Beatriz Silva Mendes, Baljit Singh

**Keywords:** abdominal closure, complications, incisional hernia, infection prevention, patient‐reported outcomes, quality of life, surgical site infection, surgical technique

## Abstract

**Background:**

The European Society of Coloproctology (ESCP) conducted a global survey capturing wound closure practices across a spectrum of surgical contexts, including clean‐contaminated and contaminated operations. The results revealed considerable variations in fascial closure techniques, suture types, and infection prevention measures, even within comparable operative scenarios. This variability highlights the urgent need for high‐quality, real‐world data to inform the development of evidence‐based, globally relevant best practices in abdominal surgery.

**Method:**

The WOLVERINE study is an international, prospective, multicenter cohort study designed to evaluate current wound closure techniques and surgical site infection (SSI) prevention strategies in adult patients undergoing general or colorectal abdominal surgery. The primary objective of this study is to investigate the relationship between wound closure materials and techniques and the development of early and late wound complications. Further objectives include quantifying the burden of wound complications on patient‐reported outcomes, health‐related quality of life, healthcare utilisation, and postoperative recovery timelines.

Participating centres include any hospital or surgical unit performing elective, expedited, or emergency abdominal, general, and colorectal operations via open, laparoscopic, or robotic approaches. This study adheres to the STROBE (Strengthening the Reporting of Observational Studies in Epidemiology) guidelines for reporting cohort studies.

**Discussion:**

The WOLVERINE cohort study will provide the first truly global, prospective portrait of abdominal wall closure practice and its short‐ and long‐term sequelae across the full spectrum of elective, expedited, and emergency general and colorectal surgery. This project bridges this fundamental knowledge gap.

## INTRODUCTION

The integrity of surgical wound closure remains a cornerstone of safe and effective abdominal surgery. Despite centuries of progress in operative techniques and wound management, tracing back to Ancient Egypt and refined through the contributions of Hippocrates, Lister, and Fleming, postoperative wound complications continue to account for a substantial proportion of surgical morbidity worldwide [1, 2, 3, 4]. Surgical site infections (SSIs), abdominal wound dehiscence, and incisional hernias persist as common and costly sequelae, leading to a diminished patient quality of life, prolonged recovery, and increased healthcare resource utilisation [5, 6, 7, 8].

Numerous randomised trials and meta‐analyses have sought to delineate the optimal strategies for wound closure and infection prevention. Investigations have explored suture materials, tension distribution methods, and adjunctive measures, such as bowel preparation and prophylactic mesh placement [9]. Patient‐level modifiable risk factors, including body mass index, smoking, and immunosuppressive medication use, have also been linked to impaired healing [10, 11, 12, 13, 14, 15]. However, complete control over these intrinsic variables is rarely possible. Consequently, the focus has shifted to standardising intraoperative techniques and environmental controls. The World Health Organisation's (WHO) global guidelines for SSI prevention represent an important step in this direction; however, their widespread adoption has been hindered by a reliance on evidence with limited strength [16].

Several landmark studies have aimed to refine the fascial closure practices. The STITCH trial demonstrated that smaller suture bites could significantly reduce the incidence of incisional hernia formation [17], whereas the PRIMA trial suggested that prophylactic onlay mesh can confer protective benefits in high‐risk laparotomies [18]. However, these findings remain controversial owing to methodological heterogeneity and limited applicability across diverse surgical populations. Systematic reviews, such as the MATCH study, have attempted to synthesise evidence across settings; however, variability in closure technique, patient selection, and quality of life endpoints continues to impede a clear consensus [19]. Even the recent European Hernia Society (EHS) guidelines, which advocate for continuous, small‐bite, slowly absorbable sutures, acknowledge a reliance on low‐ to moderate‐certainty evidence in most of their recommendations [20].

In 2023, the European Society of Coloproctology (ESCP) conducted a global survey that captured wound closure practices across various surgical contexts, including clean‐contaminated and contaminated operations. The results revealed considerable variations in fascial closure techniques, suture types, and infection prevention measures, even within comparable operative scenarios [21]. This variability highlights the urgent need for high‐quality, real‐world data to inform the development of evidence‐based, globally relevant best practices in abdominal surgery.

To address this critical gap, the Wound Closure and Surgical Site Infection Prevention Strategies in Abdominal Surgery (WOLVERINE) study was launched as a prospective, multicenter cohort study. Conducted in parallel with a structured clinical audit, WOLVERINE will generate the first large‐scale international dataset exploring the short‐ and long‐term outcomes associated with diverse wound closure and infection mitigation techniques. The study will focus on adult patients undergoing general and colorectal abdominal surgery, including elective, expedited, and emergency procedures via open, laparoscopic, or robotic approaches with a minimum incision length of 5 cm. Unlike prior trials, which were limited to narrow surgical cohorts, this cohort will reflect a broader clinical reality, including patients undergoing high‐risk and contaminated operations that are typically excluded from randomised trials.

The primary objective of the study is to investigate the relationship between wound closure materials and techniques and the development of early and late wound complications. Further objectives include quantifying the burden of wound complications on patient‐reported outcomes, health‐related quality of life, healthcare utilisation, and postoperative recovery timelines.

## METHODS

### Study design and setting

The WOLVERINE study is an international, prospective, multicenter cohort study designed to evaluate current wound closure techniques and surgical site infection (SSI) prevention strategies in adult patients undergoing general or colorectal abdominal surgery. The study is being conducted by the European Society of Coloproctology (ESCP) in collaboration with the Birmingham Centre for Observational and Prospective Studies (BiCOPS) at the University of Birmingham, which also serves as the study sponsor.

Participating centres include any hospital or surgical unit performing elective, expedited, or emergency abdominal general and colorectal operations via open, laparoscopic, or robotic approaches. Each centre is expected to enrol a minimum of 10 consecutive eligible patients within 4 weeks. The study follows the STROBE (Strengthening the Reporting of Observational Studies in Epidemiology) guidelines for cohort study reporting [22, 23].

### Participants

Eligible patients include adults aged 18 years or older undergoing abdominal surgery with a minimum incision length of 5 cm, including extraction sites. Patients may undergo elective, expedited (within 2 weeks), or emergency surgery. The procedures must fall within the scope of general or colorectal surgery. Informed consent will be required for participation in the long‐term cohort study. Patients undergoing purely hernia repair procedures (e.g. inguinal, umbilical, incisional or femoral), stoma reversals without additional laparotomy, cytoreductive surgery with HIPEC, or those who lack access to email or smart devices are excluded from participation. Each patient is enrolled only once at the time of their index operation.

### Patient identification and consent

Potential participants are identified preoperatively through outpatient clinics, operative scheduling lists, or at the point of decision for surgery, including intraoperative or postoperative identification of patients. Eligible patients will be provided with a patient information sheet (PIS) and a verbal explanation by trained personnel (surgeons, trainees or nurses). Those willing to participate will be issued a unique REDCap ID and a secure link to access the digital consent platform.

Consent is obtained electronically via the REDCap system, permitting the remote completion of follow‐up questionnaires at predefined time points. Patients consent specifically to receive follow‐up requests via email or SMS for up to 1 year postoperatively. Non‐participation in the cohort component does not affect their clinical care or inclusion in the 30‐day clinical audit.

### Data collection and follow‐up

Centres must recruit a minimum of 10 consecutive eligible patients within a 4‐week window and achieve at least 95% data completeness; those unable to meet these thresholds are offered participation in the parallel audit only. Study start‐up occurs within 1 month of local approval, and centres are expected to commence enrolment within 3 months. Data are collected at baseline, 30 days postoperatively, and at subsequent patient‐reported outcome (PRO) intervals (30, 60 and 90 days, 6 and 12 months). The initial dataset includes detailed surgical characteristics, including the type of surgery, operative urgency, contamination classification, incision type and length, wound closure materials and techniques, use of prophylactic mesh, and intraoperative SSI prevention measures.

The surgical team records clinical outcomes up to 30 days from routine medical records. These include SSI, wound dehiscence, surgical site occurrences (excluding SSI) (SSOs), reoperation, interventional radiology procedures, unplanned readmissions, intensive care unit (ICU) admissions, and 30‐day mortality. No study‐specific clinical visits are required for this study.

Beyond 30 days, data are exclusively collected from patients via online questionnaires. These tools assess wound healing, SSI burden, return to function, patient satisfaction, hernia development, and overall quality of life. Instruments that will be used include the Bluebelle Wound Healing Questionnaire (30 days), EQ‐5D‐5L (at all time points), Patient‐Reported Wound Recovery PROM (30, 60 and 90 days), ESCP Incisional Hernia PROM (90 days, 6 months, 1 year), and HerQles Questionnaire (12 months).

### Outcome definitions

The primary outcomes are early and late abdominal wound failure. Early failure includes SSI, SSO, and wound dehiscence that occur within 30 days of surgery, as defined by the CDC criteria and validated classifications [24, 25]. Late wound failure refers to the formation of an incisional hernia or infection in the presence of prosthetic material within 1 year postoperatively [26, 27, 28]. The secondary outcomes include healthcare utilisation, such as hospital stays, outpatient visits, and patient‐incurred costs.

### Data management and confidentiality

All data will be stored securely on the REDCap platform hosted by BiCOPS. Only de‐identified data will be shared between sites and the central coordinating centre. Each site retains a local linkage file to map REDCap IDs to patients for internal auditing purposes. Patient contact information is stored separately and securely, and it is deleted after the final follow‐up. A data completeness rate of at least 95% is expected for each site. Sites that fail to meet quality thresholds or exhibit non‐compliance may be subject to corrective action or suspension. Monitoring will be conducted via automated data validation checks and a central review of case report forms.

### Statistical analysis

Descriptive statistics will be used to summarise the baseline characteristics, wound closure techniques, SSI prevention practices, and incidence of short‐ and long‐term outcomes. Missing data and loss to follow‐up will be addressed using appropriate methods, such as inverse‐probability weighting or survival analysis (e.g. Cox proportional hazards models), depending on the empirical distribution of follow‐up completeness. Sensitivity analyses will be used to assess the robustness of the findings across various imputation strategies.

Subgroup analyses are pre‐planned and will explore outcome variation by geographic region (Europe vs. non‐Europe), hospital size, surgical volume, income classification, operative urgency, contamination status, surgical approach (open vs. minimally invasive), patient age, frailty, operating surgeon's training level, and specialisation. A cost–benefit analysis will be performed using reported healthcare resource utilisation and standardised economic parameters.

### Ethical considerations

The WOLVERINE study received ethical approval from the appropriate review boards, with each participating site required to obtain local approval. As a non‐interventional observational study, there are no additional clinical risks beyond those associated with the standard of care. Participation is voluntary, and patients may withdraw at any time without any consequences. No interventions are mandated or withheld.

### Study oversight

The study is centrally coordinated by the University of Birmingham. A dedicated Study Management Group (SMG), comprising the chief investigator, core investigators, statisticians, and data managers, will provide operational oversight. An International Advisory Committee (IAC) provides strategic guidance, ensures global relevance, and facilitates the dissemination of results. The study sites are led by local principal investigators and supported by multidisciplinary research teams.

## DISCUSSION

The WOLVERINE cohort study will provide the first truly global, prospective portrait of abdominal wall closure practice and its short‐ and long‐term sequelae across the full spectrum of elective, expedited, and emergency general and colorectal surgery. This project bridges a fundamental knowledge gap left by previous randomised trials and meta‐analyses, which seldom extend beyond early morbidity or include patient‐centred endpoints [17, 18, 19, 29]. This breadth of follow‐up is crucial: superficial or fascial failure in the early postoperative period is a recognised precursor of incisional hernia, a complication that can undermine the quality of life for years and consume substantial healthcare resources [5, 8, 27].

The two landmark trials that shaped the current guidelines, STITCH and PRIMA, demonstrated the benefits of a small‐bite continuous technique and prophylactic mesh placement, respectively [17, 18]. However, both were limited to elective, largely clean, or clean‐contaminated laparotomies and excluded many high‐risk scenarios routinely faced by colorectal and emergency surgeons. Subsequent systematic reviews have reiterated the potential superiority of small bites and slowly absorbable sutures but have also highlighted pervasive heterogeneity, underrepresentation of contaminated surgery, and sparse quality‐of‐life data [6, 19]. WOLVERINE directly addresses these deficiencies by enrolling an unselected international cohort—including those undergoing procedures that involve the use of ostomy bags—and by prospectively linking early events to late abdominal wall failure, incisional hernia, and patient‐centred metrics, such as quality of life and patient‐reported outcomes. In doing so, the study will benchmark contemporary practice against WHO recommendations that remain graded ‘low’ or ‘moderate’ for much of their evidence base [24].

Several features enhance the validity of the WOLVERINE. First, the network spans more than 70 countries and all income strata, ensuring that the results will be generalisable across diverse healthcare settings. Second, the mandatory consecutive enrolment of at least 10 patients per site within a pre‐specified window, coupled with routine scrutiny of recruitment, mitigates selection bias. Third, granular intraoperative documentation of bite size, suture type, and adjunctive measures provides valuable data for administrative databases. Fourth, hierarchical modelling planned a priori will account for centre‐level clustering and allow robust exploration of contextual effects, such as resource availability or dominant operative approach, on wound outcomes. Finally, remote electronic capture of PROMs minimises patient burden and permits multinational participation without the need for a costly research infrastructure.

The observational nature of the cohort precludes causal inference; unmeasured confounding and indication bias remain possible, despite multivariable adjustment and stratified analyses. Nonetheless, the rich covariates of this study should attenuate residual confounding. Loss to follow‐up, especially beyond 90 days, may threaten the validity of late outcome estimation. Inverse‐probability weighting and sensitivity analyses will be used to evaluate the impact of attrition. Variability in PROM completion across cultures and literacy levels is another concern, but translations and automated reminders are designed to maximise response rates. Misclassification of incisional hernias is possible when the diagnosis relies on clinical rather than radiological assessment. The use of an incisional hernia‐specific PROM developed by ESCP investigators is expected to improve ascertainment [26, 28].

By quantifying real‐world associations between closure technique, SSI‐prevention bundles, and wound failure, WOLVERINE will help clinicians and health‐system leaders with granular evidence to refine local protocols. Should the study confirm that small‐bite continuous closure confers a consistent advantage across contamination classes and operative approaches, the global uptake of this relatively low‐cost intervention could be accelerated. Conversely, if prophylactic mesh emerges as beneficial only in well‐defined high‐risk subgroups, targeted rather than universal deployment would avoid unnecessary expenditures and implant‐related morbidity. In parallel, health economic analyses derived from length of stay, readmission, and reoperation data will clarify the true cost burden of early and late wound failure, informing reimbursement strategies [6].

The study is explicitly designed to support a future pragmatic, randomised, adaptive trial that will test the most promising interventions under conditions that mirror those of the WOLVERINE. The baseline event rates and variance estimates of the present study will efficiently power the trial, whereas the established ESCP network can pivot seamlessly to an interventional platform. Moreover, the anonymised dataset will form the backbone of an interactive education environment, enabling iterative audit‐feedback cycles that have previously driven quality improvement in colorectal surgery.

## CONCLUSION

WOLVERINE represents a collaboration aimed at resolving one of the oldest challenges in surgery. By coupling meticulous, surgeon‐reported perioperative data with longitudinal patient perspectives, this study will lay the groundwork for future interventional studies to enhance the standardisation and possibly stratification of abdominal wall closure and surgical site infection prevention techniques. The findings aim to improve surgical knowledge, impact global clinical practice, and inform the development of international guidelines, ultimately improving the long‐term well‐being of patients undergoing abdominal surgery worldwide.

## FUNDING INFORMATION

This study is supported by Ethicon.

## CONFLICT OF INTEREST STATEMENT

The authors declare no conflicts of interest.

## ETHICS STATEMENT

Research Ethics Committee (REC) and the institutional review boards of participating centres are responsible for approving the study protocol as required. Local principal investigators are responsible for obtaining clinical audit, institutional review board, or ethical approval according to the regional and national regulations.

## PROTOCOL VERSION

V1, August 2025.

## ISRCTN REGISTRATION

55324472.

## Data Availability

Data sharing not applicable to this article as no datasets were generated or analysed during the current study.
